# Associations of estimated plasma volume status with 30-day mortality and 1-year mortality in patients with intracerebral hemorrhage: a study of the MIMIC-IV database and the hospital information system

**DOI:** 10.3389/fneur.2025.1548064

**Published:** 2025-04-01

**Authors:** Yongze Shen, Yingjie Shen, Yaolou Wang, Renjie Hu, Hangjia Xu, Yuyang Feng, Yang Yang, Xiangtong Zhang, Hongsheng Liang

**Affiliations:** Department of Neurosurgery, The First Affiliated Hospital of Harbin Medical University, Harbin, Heilongjiang, China

**Keywords:** ePVS, intracerebral hemorrhage, 30-day mortality, 1-year mortality, MIMIC-IV database

## Abstract

**Aim:**

This study aims to investigated the associations between estimated plasma volume status (ePVS) and 30-day and 1-year mortality in intracerebral hemorrhage (ICH) patients, providing insights into the management in ICH.

**Methods:**

Data of adult ICH patients were extracted from both the Medical Information Mart for Intensive Care IV (MIMIC-IV) database and the Hospital Information System (HIS) in this retrospective cohort study. Univariate and multivariate Cox regression analyses, and restricted cubic spline plots (RCS) were conducted to explore the associations between ePVS levels and both 30-day and 1-year mortality, with hazard ratios (HR) and 95% confidence intervals (CI) used for evaluation. Subgroup analyses were performed to further investigate these associations.

**Results:**

Among 2,512 eligible patients from the MIMIC-IV database, 655 (26.07%) died within 30 days, with 1,254 (49.92%) had died by the 1-year follow-up. After adjusting for covariates, elevated ePVS was independently associated with both 30-day mortality (HR = 1.05, 95%CI: 1.01–1.09) and 1-year mortality (HR = 1.09, 95% CI: 1.06–1.13). Compared to patients with ePVS levels of [4.63–5.79), those with ePVS levels ≥5.79 had a higher risk of 30-day mortality (HR: 1.36, 95%CI: 1.12–1.64) and 1-year mortality (HR = 1.24, 95% CI: 1.08–1.42). Among 515 eligible patients from the HIS, 132 (25.60%) died within 30 days, with 288 (55.90%) mortality observed at 1-year follow-up. After adjusting for covariates, elevated ePVS was independently associated with both 30-day mortality (HR = 1.33, 95%CI: 1.23–1.43) and 1-year mortality (HR = 1.26, 95% CI: 1.18–1.35). Comparing to patients with ePVS levels of [4.63–5.79), those with ePVS levels of ≥5.79 had a higher risk of 30-day mortality (HR:2.21, 95%CI: 1.48–3.30) and 1-year mortality (HR = 2.75, 95% CI: 2.04–3.72). Additionally, subgroup analyses demonstrated that ePVS was significantly associated with 30-day mortality or 1-year mortality derived from MIMIC-IV and HIS in most subgroups (*p* < 0.05). And RCS analysis indicates that, whether using MIMIC-IV or HIS data, ePVS was linearly associated with 30-day or 1-year mortality.

**Conclusion:**

Higher ePVS levels may be a potential risk factor for 30-day and 1-year mortality in ICH patients, suggesting that timely monitoring and stabilization of ePVS could improve prognosis in this population. However, further studies are needed to validate these fingings.

## 1 Introduction

In recent years, intracerebral hemorrhage (ICH) has caused a significant disease burden worldwide (1, 2) and is an important cause of short-term mortality in intensive care units (ICUs) (3, 4). Therefore, it is crucial to identify risk factors related to mortality in ICH to improve patient prognosis.

In patients with critical brain injury, both hypovolemia and hypervolemia can lead to secondary brain injury (5). Elevated plasma volume has been reported to be associated with adverse outcomes in patients with acute ischemic stroke who received endovascular therapy, possibly due to overactivation of the renin-angiotensin-aldosterone system, hemodynamic instability, and changes in blood pressure (6). Studies have found that patients with a positive fluid balance have longer hospital stays and an increased risk of acute kidney injury (AKI) (7, 8). However, few studies have investigated the relationship between baseline plasma volume status (PVS) and prognosis in patients with ICH. Additionally, the estimated plasma volume status (ePVS) is based on calculations of hemoglobin (Hb) and hematocrit (HCT) and is a low-cost, rapid quantification method for monitoring plasma volume fluctuations (9). A higher ePVS level has been associated with an increased risk of AKI in patients who underwent cardiac surgery, as well as an increased mortality risk in patients with heart failure or sepsis (10–12). However, no studies have explored the association between ePVS and the risk of short-term mortality in patients with ICH.

In this study, we aimed to investigate the association between ePVS and 30-day and 1-year mortality in patients with ICH, using data extracted from the Medical Information Mart for Intensive Care IV (MIMIC-IV) database and the Hospital Information System (HIS) of the First Affiliated Hospital of Harbin Medical University, and to provide a reference for prognostic risk identification and fluid management in this population.

## 2 Methods

### 2.1 Study design and participants

In this retrospective cohort study, data of patients with ICH were extracted from two sources. Firstly, data were obtained from the MIMIC-IV database from 2008 to 2022. The MIMIC database is published by the Massachusetts Institute of Technology (MIT, Cambridge, MA, United States), Philips Medical, and Beth Israel Deaconess Medical Center (BIDMC, Boston, MA, United States). The database collects and organizes clinical diagnosis and treatment information on more than 40,000 ICU patients, who have been predominantly white, since 2001. More detailed information about this database is provided elsewhere.^1^

**Figure 1:**
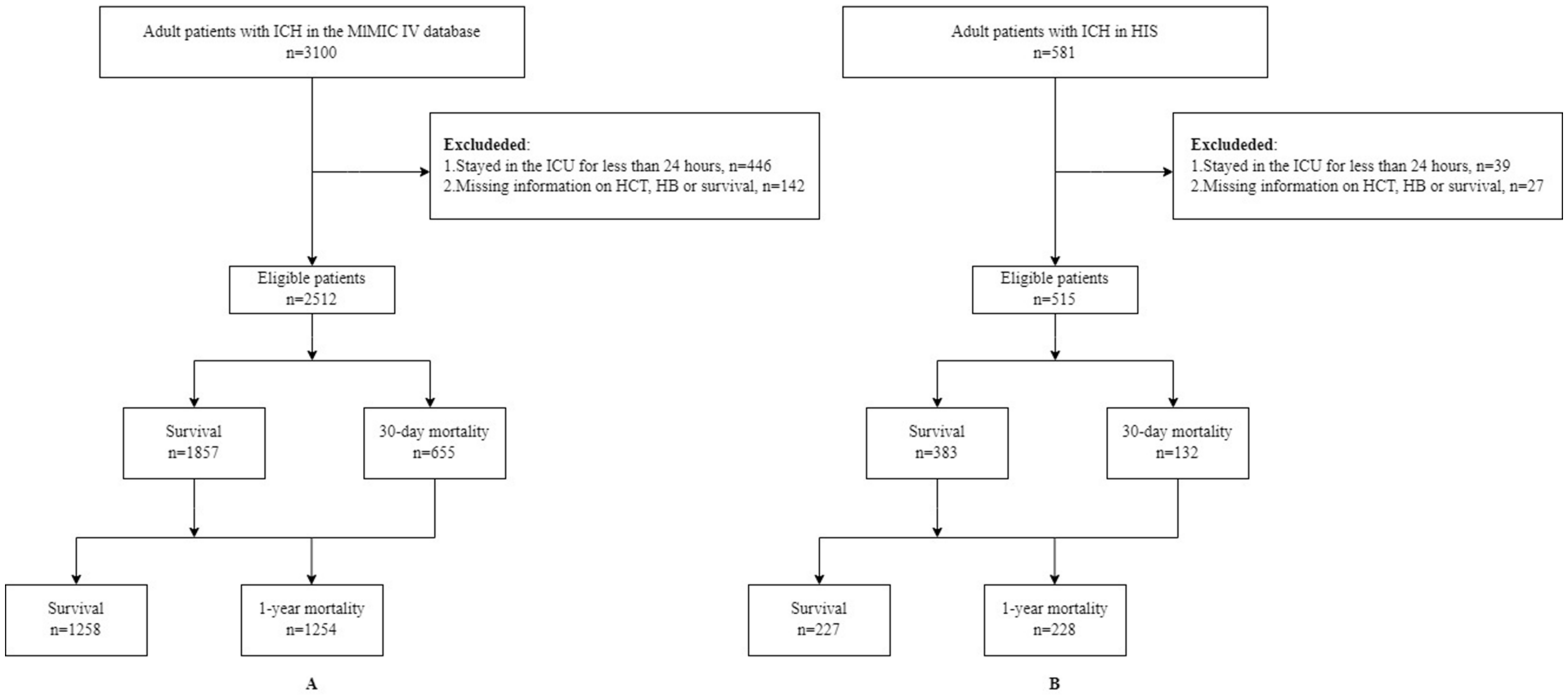
Flowchart of the study process. **(A)** MIMIC-IV. **(B)** HIS.

We initially included 3,100 adult patients diagnosed with ICH from the MIMIC-IV database. The diagnosis of ICH was based on the International Classification of Diseases (ICD), 9th Edition (codes beginning with 431) or 10th Edition (codes beginning with I61) (13). The exclusion criteria were as follows: (1) hospitalization in the ICU for less than 24 h, (2) no measurement of hematocrit (HCT) and hemoglobin (Hb) at ICU admission, and (3) missing information on survival, and (4) concurrent ICD codes for traumatic brain injury (TBI), arteriovenous malformations (AVM), aneurysmal rupture, coagulopathy, or malignancy-related hemorrhage. Finally, 2,512 patients were included in the study (Figure 1A). The MIMIC database was approved by the Institutional Review Boards (IRBs) of BIDMC and MIT. Since patient data were de-identified and the database was publicly available, ethical approval was waived by the IRB of our hospital.

Secondly, data were also extracted from the HIS of the First Affiliated Hospital of Harbin Medical University, with the data spanning from January 2022 to December 2024. A total of 581 patients with ICH were initially identified from the hospital records during this period. The inclusion and exclusion criteria were the same as those for the MIMIC-IV database. After applying these criteria, 515 patients were finally selected for the study (Figure 1B). The research protocol has been reviewed and approved by the Ethics Committee of the First Affiliated Hospital of Harbin Medical University. Informed consent was obtained from patients or their legal representatives.

### 2.2 Calculation of ePVS

The ePVS was calculated according to the Strauss-derived Duarte formula (9): ePVS = [100−HCT (%)]/Hb (g/dL). HCT and Hb levels were measured within 24 h of admission to the ICU. In this study, ePVS was divided into three categories based on tertiles: <4.63, [4.63–5.79), and ≥ 5.79.

We also used the KH-ePVS to analyze the association between ePVS and 30-day mortality in a sensitivity analysis (14). Specifically, KH-ePVS was calculated using the following formula: KH-ePVS = [(aPV−iPV)/iPV] × 100%. aPV = (1−HCT) × [a + b × weight (kg)], where the adjustment factors were a = 1,530 in men and 864 in women, and b = 41 in men and 47.9 in women. The iPV = c × weight (kg), where the adjustment factor for c was 39 in men and 40 in women.

### 2.3 Covariables selection

We also selected variables that could potentially be covariates associated with 30-day and 1-year mortality in patients with ICH from the MIMIC-IV database and the HIS, including age, gender, race (only MIMIC-IV), ICU type, cerebral infarction, diabetes mellitus (DM), heart failure (HF), acute kidney injury (AKI), sepsis, heart rate (HR), mean arterial pressure (MAP), respiratory rate (RR), temperature, SpO2, the Sequential Organ Failure Assessment (SOFA) score, the Glasgow Coma Scale (GCS), the Charlson Comorbidity Index (CCI), white blood cell (WBC) count, platelets, creatinine (Cr), blood urea nitrogen (BUN), glucose, calcium (Ca), anion gap (AG), prothrombin time (PT), urine output, brain surgery, renal replacement therapy (RRT), mechanical ventilation, vasopressors, mannitol, furosemide, transfusion, and *β*-blockers. Information on these variables was extracted only when patients were admitted to the ICU for the first time. AKI was defined according to the Kidney Disease: Improving Global Outcomes (KDIGO) criteria, which includes an increase in serum creatinine by ≥0.3 mg/dL within 48 h, an increase to ≥1.5 times baseline within 7 days, or urine output <0.5 mL/kg/h for 6 h. Cerebral infarction was defined as a history of ischemic stroke confirmed by clinical assessment and neuroimaging (CT or MRI). HF was determined based on clinical diagnosis supported by echocardiographic findings, elevated brain natriuretic peptide (BNP) levels, or clinical signs such as pulmonary congestion or peripheral edema. Sepsis was defined according to the Sepsis-3 criteria, requiring suspected or confirmed infection plus a SOFA score ≥ 2. Brain surgery included craniotomy, minimally invasive procedures, decompressive craniectomy, burr hole drainage, and external ventricular drainage (EVD), as recorded in the procedural data. Mechanical ventilation was defined as the use of endotracheal intubation with invasive positive pressure ventilation. Vasopressors included norepinephrine, dopamine, epinephrine, or vasopressin administered during ICU stay. Transfusion included red blood cell (RBC), plasma, and platelet transfusions, while *β*-blocker use was identified through medication records. Similarly, the diagnosis of complications and therapies was based on ICD-9 and ICD-10 codes.

### 2.4 Outcomes and follow-up duration

The study outcome was 30-day and 1-year mortality. The MIMIC-IV database and the HIS was followed by information from electronic medical charts and hospital department records, which were supplemented by contacting patients, their family members, attending healthcare workers, or family physicians by phone. In this study, follow-up ended when the patient died in the hospital or one year after ICU admission.

### 2.5 Statistical analysis

Normally distributed data were described using mean ± standard deviation (mean ± SD). Analysis of variance (ANOVA) was used to compare patient characteristics among different ePVS level groups. Non-normally distributed data were described using the median and interquartile range [M (Q1, Q3)], and the Kruskal-Wallis H test was used for comparison. The distribution of categorical data was described using frequency and composition ratios [N (%)], and the chi-square test (χ^2^) was employed for comparison.

Univariate Cox regression and stepwise regression analyses were used to screen for covariates associated with 30-day and 1-year mortality in patients with ICH. Variables with *p* < 0.05 in univariable analysis were considered for inclusion in the multivariable model. Univariable and multivariable Cox regression analyses were conducted to investigate the association between ePVS and 30-day mortality. The association was evaluated using hazard ratios (HRs) and 95% confidence intervals (CIs). Model 1 was unadjusted. Model 2 was adjusted for selected covariates, including age, race (only MIMIC-IV), ICU type, HR, SpO2, SOFA, CCI, platelets, glucose, AG, urine output, mechanical ventilation, vasopressors, mannitol, and *β*-blockers. The Kaplan–Meier (KM) curve was plotted to reflect the survival probability in patients with ICH across different ePVS level groups over the follow-up period. Additionally, subgroup analyses of age, GCS score, SOFA score, AKI, sepsis, and HF were performed to assess the association between ePVS and 30-day and 1-year mortality. A two-sided *p* value <0.05 was considered statistically significant.

Regarding sensitivity analysis, we investigated the association between KH-ePVS and 30-day and 1-year mortality in patients with ICH. First, since the calculation of KH-ePVS was based on weight, we excluded patients with missing weight information (the final sample size was 1,637). Covariates associated with 30-day and 1-year mortality in patients with ICH were screened using univariate Cox regression analysis and stepwise regression. The association between KH-ePVS and 30-day and 1-year mortality was evaluated using univariate and multivariate Cox regression analyses. Model 1 was the unadjusted model, and Model 2 was adjusted for age, ICU type, AG, race, vasopressors, mannitol, SOFA, CCI, glucose, *β*-blockers, mechanical ventilation, and urine output. And restricted cubic spline plots (RCS) was also conducted to explore the potential nonlinear association between ePVS and 30-day and 1-year mortality.

Variables with missing data were interpolated using the multiple imputation method (Table S1) (15). Sensitivity analysis of ICH patient characteristics before and after the interpolation of missing data is shown in Table S2. Statistical analyses were performed using Python 3.9.12 (Python Software Foundation, Delaware, United States) and R version 4.2.3 (Institute for Statistics and Mathematics, Vienna, Austria).

## 3 Results

### 3.1 Characteristics of participants

The characteristics of the patients with ICH in the different ePVS level groups are shown in Table 1. Among the total population, 655 (26.07%) patients died within 30 days, with 1,254 (49.92%) mortality observed at 1-year follow-up. The average age of the patients was 66 years, and 1,152 (45.86%) were female. In the ePVS level ≥ 5.79 group, patients who died within 30 days after ICU admission represented the largest proportion, followed by those in the ePVS level [4.63–5.79) group and the ePVS level < 4.63 group (33.49% vs. 24.03% vs. 20.75%). In addition, race, ICU type, DM, HF, AKI, sepsis, HR, MAP, SpO2, SOFA, GCS, CCI, WBC, platelets, Cr, BUN, Ca, AG, PT, RRT, mechanical ventilation, vasopressor use, furosemide, and transfusion were all significantly different among the three groups (all *p* < 0.05).

**Table 1:** Characteristics of ICH patients in different ePVS level groups.

Variables	Total (*n* = 2,512)	ePVS levels			Statistics	*P*
		<4.63 (*n* = 829)	[4.63–5.79) (*n* = 853)	≥5.79 (*n* = 830)		
Age, years, mean ± SD	66.39 ± 15.37	63.15 ± 14.86	68.23 ± 15.22	67.73 ± 15.52	*F* = 28.258	**<0.001**
Gender, *n* (%)					χ^2^ = 208.214	**<0.001**
Female	1,152 (45.86)	215 (25.93)	442 (51.82)	495 (59.64)		
Male	1,360 (54.14)	614 (74.07)	411 (48.18)	335 (40.36)		
Race, *n* (%)					χ^2^ = 36.617	**<0.001**
White	1,460 (58.12)	469 (56.57)	539 (63.19)	452 (54.46)		
Black	249 (9.91)	58 (7.00)	76 (8.91)	115 (13.86)		
Others	803 (31.97)	302 (36.43)	238 (27.90)	263 (31.69)		
ICU types, *n* (%)					χ^2^ = 69.313	**<0.001**
Neuro intermediate/stepdown	916 (36.46)	353 (42.58)	300 (35.17)	263 (31.69)		
SICU	1,397 (55.61)	433 (52.23)	510 (59.79)	454 (54.70)		
Others	199 (7.92)	43 (5.19)	43 (5.04)	113 (13.61)		
Cerebral infarction, *n* (%)					χ^2^ = 1.528	0.466
No	2,348 (93.47)	771 (93.00)	794 (93.08)	783 (94.34)		
Yes	164 (6.53)	58 (7.00)	59 (6.92)	47 (5.66)		
DM, *n* (%)					χ^2^ = 11.682	**0.003**
No	1854 (73.81)	640 (77.20)	634 (74.33)	580 (69.88)		
Yes	658 (26.19)	189 (22.80)	219 (25.67)	250 (30.12)		
HF, *n* (%)					χ^2^ = 19.776	**<0.001**
No	2,158 (85.91)	725 (87.45)	756 (88.63)	677 (81.57)		
Yes	354 (14.09)	104 (12.55)	97 (11.37)	153 (18.43)		
AKI, *n* (%)					χ^2^ = 7.054	**0.029**
No	915 (36.43)	307 (37.03)	334 (39.16)	274 (33.01)		
Yes	1,597 (63.57)	522 (62.97)	519 (60.84)	556 (66.99)		
Sepsis, *n* (%)					χ^2^ = 13.956	**<0.001**
No	1836 (73.09)	620 (74.79)	648 (75.97)	568 (68.43)		
Yes	676 (26.91)	209 (25.21)	205 (24.03)	262 (31.57)		
HR, bpm, Mean ± SD	82.87 ± 17.64	83.44 ± 18.23	81.15 ± 16.40	84.08 ± 18.14	*F* = 6.471	**0.002**
MAP, mmHg, Mean ± SD	96.23 ± 17.18	99.73 ± 16.48	96.41 ± 17.05	92.55 ± 17.27	*F* = 37.351	**<0.001**
RR, insp/min, Mean ± SD	18.64 ± 5.23	18.83 ± 5.39	18.45 ± 5.09	18.64 ± 5.21	*F* = 1.094	0.335
Temperature, °C, Mean ± SD	36.84 ± 0.65	36.84 ± 0.60	36.85 ± 0.61	36.83 ± 0.74	*F* = 0.271	0.762
SpO_2_, %, Mean ± SD	97.38 ± 3.31	97.07 ± 2.72	97.36 ± 4.02	97.69 ± 2.98	*F* = 7.403	**<0.001**
SOFA, M (Q_1_, Q_3_)	1.00 (0.00, 2.00)	0.00 (0.00, 1.00)	1.00 (0.00, 2.00)	1.00 (0.00, 2.00)	W = 55.798	**<0.001**
GCS, Mean ± SD	13.99 ± 2.04	14.14 ± 1.76	14.01 ± 1.97	13.82 ± 2.35	*F* = 5.169	**0.006**
CCI, M (Q_1_, Q_3_)	3.00 (2.00, 5.00)	3.00 (1.00, 4.00)	3.00 (2.00, 4.00)	4.00 (2.00, 6.00)	W = 108.174	**<0.001**
WBC, K/uL, M (Q_1_, Q_3_)	10.25 (7.90, 13.20)	10.80 (8.30, 13.50)	10.00 (8.00, 12.80)	9.95 (7.70, 13.10)	W = 14.869	**<0.001**
Platelet, K/uL, M (Q_1_, Q_3_)	206.00 (161.00, 259.00)	210.00 (172.00, 256.00)	207.00 (163.00, 255.00)	199.00 (146.00, 265.00)	W = 11.843	**0.003**
Cr, mg/dL, M (Q_1_, Q_3_)	0.90 (0.70, 1.10)	0.90 (0.80, 1.10)	0.90 (0.70, 1.10)	0.90 (0.70, 1.30)	W = 14.514	**<0.001**
BUN, mg/dL, M (Q_1_, Q_3_)	16.00 (12.00, 22.00)	15.00 (12.00, 20.00)	16.00 (12.00, 21.00)	18.00 (13.00, 28.00)	W = 49.872	**<0.001**
Glucose, mg/dL, M (Q_1_, Q_3_)	129.00 (107.00, 160.00)	127.00 (108.00, 158.00)	130.00 (108.00, 158.00)	130.50 (106.00, 162.00)	W = 0.561	0.755
Ca, mg/dL, Mean ± SD	8.73 ± 0.73	8.92 ± 0.62	8.75 ± 0.66	8.54 ± 0.85	*F* = 58.342	**<0.001**
AG, Mean ± SD	16.31 ± 3.91	16.79 ± 3.42	16.04 ± 3.55	16.12 ± 4.62	*F* = 9.376	**<0.001**
PT, sec, M (Q_1_, Q_3_)	12.60 (11.80, 14.00)	12.30 (11.60, 13.50)	12.50 (11.70, 13.70)	13.10 (12.00, 15.10)	W = 85.404	**<0.001**
Urine output, ml, M (Q_1_, Q_3_)	1550.00 (990.00, 2278.00)	1585.00 (1050.00, 2305.00)	1550.00 (1008.00, 2335.00)	1527.50 (900.00, 2215.00)	W = 5.276	0.072
Brain surgery, *n* (%)					χ^2^ = 4.326	0.115
No	2,393 (95.26)	789 (95.17)	822 (96.37)	782 (94.22)		
Yes	119 (4.74)	40 (4.83)	31 (3.63)	48 (5.78)		
RRT, *n* (%)					χ^2^ = 57.929	**<0.001**
No	2,424 (96.50)	818 (98.67)	838 (98.24)	768 (92.53)		
Yes	88 (3.50)	11 (1.33)	15 (1.76)	62 (7.47)		
Mechanical ventilation, *n* (%)					χ^2^ = 12.733	**0.002**
No	634 (25.24)	229 (27.62)	232 (27.20)	173 (20.84)		
Yes	1878 (74.76)	600 (72.38)	621 (72.80)	657 (79.16)		
Vasopressors, *n* (%)					χ^2^ = 17.370	**<0.001**
No	2074 (82.56)	703 (84.80)	723 (84.76)	648 (78.07)		
Yes	438 (17.44)	126 (15.20)	130 (15.24)	182 (21.93)		
Mannitol, *n* (%)					χ^2^ = 2.161	0.339
No	2,322 (92.44)	759 (91.56)	797 (93.43)	766 (92.29)		
Yes	190 (7.56)	70 (8.44)	56 (6.57)	64 (7.71)		
Furosemide, *n* (%)					χ^2^ = 15.750	**<0.001**
No	2038 (81.13)	687 (82.87)	714 (83.70)	637 (76.75)		
Yes	474 (18.87)	142 (17.13)	139 (16.30)	193 (23.25)		
Transfusion, *n* (%)					χ^2^ = 221.048	**<0.001**
Non-transfusion	2,113 (84.12)	770 (92.88)	762 (89.33)	581 (70.00)		
Plasma	77 (3.07)	18 (2.17)	23 (2.70)	36 (4.34)		
Thrombocyte	80 (3.18)	19 (2.29)	25 (2.93)	36 (4.34)		
RBC	136 (5.41)	12 (1.45)	25 (2.93)	99 (11.93)		
Others	106 (4.22)	10 (1.21)	18 (2.11)	78 (9.40)		
β-blockers, *n* (%)					χ^2^ = 5.043	0.080
No	638 (25.40)	205 (24.73)	200 (23.45)	233 (28.07)		
Yes	1874 (74.60)	624 (75.27)	653 (76.55)	597 (71.93)		
30-day mortality, *n* (%)					χ^2^ = 37.750	**<0.001**
No	1857 (73.93)	657 (79.25)	648 (75.97)	552 (66.51)		
Yes	655 (26.07)	172 (20.75)	205 (24.03)	278 (33.49)		
1-year mortality, *n* (%)					χ^2^ = 110.965	**<0.001**
No	1,258 (50.10)	528 (63.70)	415 (48.70)	315 (38.00)		
Yes	1,254 (49.90)	301 (36.30)	438 (51.30)	515 (62.00)		
Follow-up time, days, M (Q_1_, Q_3_)	30.00 (25.13, 30.00)	30.00 (30.00, 30.00)	30.00 (30.00, 30.00)	30.00 (14.03, 30.00)	W = 34.001	**<0.001**

F: ANOVA, W: Kruskal-Wallis H test, χ^2^: chi-square test. The bold *p* values represented statistically significant. ICH: intracerebral hemorrhage, ePVS: estimated plasma volume status, SD: standard deviation, ICU: intensive care unit, SICU: surgical intensive care unit, DM: diabetes mellitus, HF: heart failure, AKI: acute kidney injury, HR: heart rate, MAP: mean arterial pressure, RR: respiratory rate, SOFA: the Sequential Organ-Failure Assessment, M: median, Q_1_: 1st quartile, Q_3_: 3rd quartile, GCS: the Glasgow Coma Scale, CCI: the Charlson Comorbidity Index, WBC: white blood cell, Cr: creatinine, BUN: blood urea nitrogen, Ca: calcium, AG: anion gap, PT: prothrombin time, RRT: renal replacement therapy, RBC: red blood cell.

In Table S3, higher ePVS levels were significantly associated with several variables. For example, the GCS score was significantly reduced (*p* < 0.001), the SOFA score was significantly increased (*p* = 0.043), and WBC count was significantly higher (*p* = 0.025), as was BUN (*p* = 0.029). Furthermore, the use of furosemide (Lasix) was significantly higher in the higher ePVS group (*p* = 0.043). Additionally, the higher the ePVS, the higher the 30-day and 1-year mortality rates, and these trends were statistically significant (both *p* < 0.001).

### 3.2 Association between ePVS and 30-day and 1-year mortality in patients with ICH

As shown in Table 2, after adjusting for the selected covariates, an elevated ePVS was associated with an increased risk both of 30-day and 1-year mortality. For the MIMIC-IV data, an elevated ePVS was associated with an increased risk of 30-day mortality (HR = 1.05, 95%CI: 1.01–1.09). Compared to patients with ePVS levels of [4.63–5.79), those with ePVS levels of ≥5.79 had a higher risk of 30-day mortality, with an HR (95%CI) of 1.36 (1.12–1.64). In the HIS dataset, the association was even stronger, with an HR of 1.33 (95%CI: 1.23–1.43) for ePVS. Patients with ePVS levels of ≥5.79 had a much higher risk of 30-day mortality, with an HR (95%CI) of 2.21 (1.48–3.30) compared to the reference group ([4.63–5.79)). For 1-year mortality, the results were similarly significant. In the MIMIC-IV data, ePVS was associated with a higher risk of 1-year mortality (HR = 1.09, 95%CI: 1.06–1.13). Patients with ePVS ≥5.79 had an HR (95%CI) of 1.24 (1.08–1.42). In the HIS dataset, the association was even more pronounced, with an HR for ePVS of 1.26 (95%CI: 1.18–1.35). Patients with ePVS levels of ≥5.79 had a notably higher risk of 1-year mortality, with an HR (95%CI) of 2.75 (2.04–3.72), again showing a significantly greater likelihood of mortality for those with higher ePVS.

**Table 2:** Association of ePVS with 30-day and 1-year mortality in ICH patients.

Variables	Model 1		Model 2	
	HR (95% CI)	*P*	HR (95% CI)	*P*
30-day mortality				
MIMIC-IV				
ePVS	1.11 (1.07–1.14)	**<0.001**	1.05 (1.01–1.09)	**0.012**
ePVS levels				
[4.63–5.79)	Ref		Ref	
< 4.63	0.86 (0.70–1.05)	0.139	1.00 (0.81–1.23)	0.980
≥ 5.79	1.48 (1.23–1.77)	**<0.001**	1.36 (1.12–1.64)	**0.002**
HIS				
ePVS	1.31 (1.23–1.41)	**<0.001**	1.33 (1.23–1.43)	**<0.001**
ePVS levels				
[4.63–5.79)	Ref		Ref	
< 4.63	0.60 (0.39–0.92)	0.021	0.63 (0.41–0.98)	0.040
≥ 5.79	2.24 (1.51–3.34)	**<0.001**	2.21 (1.48–3.30)	**<0.001**
1-year mortality				
MIMIC-IV				
ePVS	1.13 (1.10–1.16)	**<0.001**	1.09 (1.06–1.13)	**<0.001**
ePVS levels				
[4.63–5.79)	Ref		Ref	
< 4.63	0.66 (0.57–0.77)	**<0.001**	0.72 (0.63–0.83)	**<0.001**
≥ 5.79	1.31 (1.15–1.48)	**<0.001**	1.24 (1.08–1.42)	**0.002**
HIS				
ePVS	1.26 (1.18–1.35)	**<0.001**	1.26 (1.18–1.35)	**<0.001**
ePVS levels				
[4.63–5.79)	Ref		Ref	
< 4.63	1.09 (0.82–1.44)	0.561	1.09 (0.83–1.44)	0.534
≥ 5.79	2.76 (2.04–3.73)	**<0.001**	2.75 (2.04–3.72)	**<0.001**

The bold *P* values represented statistically significant. MIMIC-IV: Medical Information Mart for Intensive Care IV, HIS: Hospital Information System, ePVS: estimated plasma volume status, ICH: intracerebral hemorrhage, HR: hazard ratio, CI: confidence interval, Ref: reference. For 30-day mortality, Model 1: unadjusted model; Model 2: adjusted for age, race (only MIMIC-IV), ICU types, HR, SpO_2_, SOFA, CCI, platelet, glucose, AG, urine output, mechanical ventilation, vasopressors, mannitol and β-blockers. For 1-year mortality, Model 1: unadjusted model; Model 2: adjusted for age, race (only MIMIC-IV), ICU types, HR, SpO_2_, SOFA, CCI, platelet, glucose, AG, brain surgery, mechanical ventilation, vasopressors, mannitol and transfusion.

**Figure 2:**
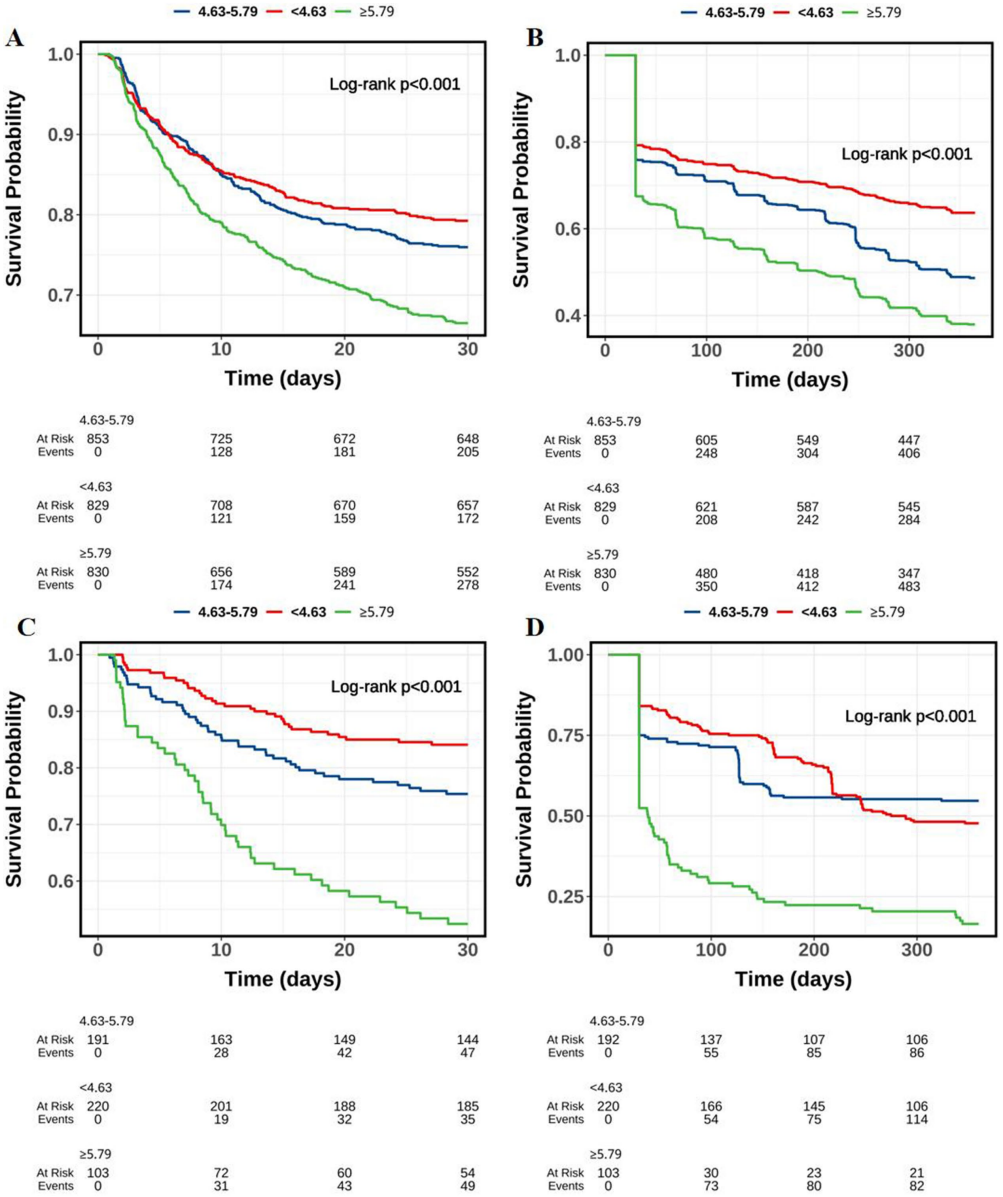
Kaplan–Meier survival curves of probability of ICH patients in different ePVS level groups along with the follow-up time. **(A)** MIMIC-IV (30-day mortality); **(B)** MIMIC-IV (1-year mortality); **(C)** HIS (30-day mortality); **(D)** HIS (1-year mortality).

Similarly, as shown in Figure 2, along with the follow-up time, the survival probability of patients in the ePVS level of ≥5.79 group showed the largest decline, followed by the ePVS level of [4.63–5.79) group and the ePVS level of <4.63 group (*p* < 0.05).

### 3.3 Associations between ePVS levels and 30-day and 1-year mortality in different subgroups

In the results of Table S4 (MIMIC-IV), the positive association between ePVS and 30-day mortality was primarily observed in multiple subgroups. In patients aged ≥60 years, ePVS was significantly associated with 30-day mortality (HR = 1.05, 95%CI: 1.01–1.11), and patients with ePVS levels ≥5.79 had a significantly increased risk of death (HR = 1.28, 95%CI: 1.04–1.58). In patients with a GCS score ≥ 13, the association between ePVS and mortality was also significant (HR = 1.05, 95%CI: 1.01–1.09), and patients with ePVS levels ≥5.79 had a significantly increased risk of death (HR = 1.35, 95%CI: 1.10–1.66). In patients with a SOFA score < 1, ePVS was significantly associated with 30-day mortality (HR = 1.12, 95%CI: 1.04–1.21), and although the risk of death for patients with ePVS levels ≥5.79 was approaching significance (HR = 1.29, 95% CI: 0.95–1.74), it remained noteworthy. In non-AKI patients, ePVS was significantly associated with mortality (HR = 1.14, 95%CI: 1.02–1.28), and patients with ePVS levels ≥5.79 had a significantly increased risk of death (HR = 1.58, 95%CI: 1.11–2.24). Furthermore, in non-sepsis patients, ePVS was significantly associated with 30-day mortality (HR = 1.06, 95%CI: 1.01–1.11), and patients with ePVS levels ≥5.79 had a significantly increased risk of death (HR = 1.42, 95%CI: 1.13–1.79). Finally, in non-heart failure patients, ePVS was significantly associated with 30-day mortality (HR = 1.06, 95%CI: 1.01–1.10), and patients with ePVS levels ≥5.79 had a significantly increased risk of death (HR = 1.46, 95%CI: 1.18–1.80).

In the results of Table S5 (MIMIC-IV), the positive association between ePVS and 1-year mortality was observed in several subgroups. In patients aged ≥60 years, ePVS was significantly associated with 1-year mortality (HR = 1.11, 95%CI: 1.07–1.15), and patients with ePVS levels ≥5.79 had a significantly increased risk of death (HR = 1.25, 95%CI: 1.08–1.44). In patients with a GCS score ≥ 13, ePVS was significantly associated with mortality (HR = 1.05, 95%CI: 1.01–1.09), and patients with ePVS levels ≥5.79 had a significantly increased risk of death (HR = 1.25, 95%CI: 1.08–1.44). In patients with a SOFA score < 1, ePVS was significantly associated with 1-year mortality (HR = 1.13, 95%CI: 1.08–1.19), and patients with ePVS levels ≥5.79 had a significantly increased risk of death (HR = 1.27, 95%CI: 1.04–1.54). In non-AKI patients, ePVS was significantly associated with 1-year mortality (HR = 1.15, 95%CI: 1.07–1.23), and patients with ePVS levels ≥5.79 had a significantly increased risk of death (HR = 1.36, 95%CI: 1.09–1.70). Similarly, in AKI patients, ePVS was significantly associated with mortality (HR = 1.09, 95%CI: 1.06–1.12), and patients with ePVS levels ≥5.79 also had a significantly increased risk of death (HR = 1.20, 95%CI: 1.02–1.41). In non-sepsis patients, ePVS was significantly associated with 1-year mortality (HR = 1.11, 95%CI: 1.08–1.15), and patients with ePVS levels <4.63 had a significantly lower risk of death (HR = 0.68, 95%CI: 0.57–0.81). In patients with sepsis, ePVS was significantly associated with mortality (HR = 1.08, 95%CI: 1.02–1.13), and patients with ePVS levels <4.63 had a significantly lower risk of death (HR = 0.73, 95%CI: 0.54–0.98). Lastly, in non-heart failure patients, ePVS was significantly associated with 1-year mortality (HR = 1.10, 95%CI: 1.07–1.14), and patients with ePVS levels ≥5.79 had a significantly increased risk of death (HR = 1.31, 95%CI: 1.14–1.51).

In Table S6 (HIS), the positive association between ePVS and 30-day mortality was observed across multiple subgroups. In patients aged ≥60 years, ePVS was significantly associated with 30-day mortality (HR = 1.55, 95%CI: 1.35–1.79), and those with ePVS levels ≥5.79 had a significantly higher risk of death (HR = 2.25, 95%CI: 1.40–3.63). In patients with a GCS score < 13, ePVS was significantly associated with mortality (HR = 1.22, 95%CI: 1.13–1.31), and those with ePVS levels ≥5.79 had a significantly higher risk of death (HR = 2.23, 95%CI: 1.44–3.45). In patients with a SOFA score < 1, ePVS was strongly associated with 30-day mortality (HR = 1.56, 95%CI: 1.28–1.91), with those having ePVS levels ≥5.79 showing a significantly increased risk of death (HR = 1.90, 95%CI: 1.01–3.56). In non-AKI patients, ePVS was significantly associated with 30-day mortality (HR = 1.14, 95%CI: 1.02–1.28), and those with ePVS levels ≥5.79 showed a significantly increased risk of death (HR = 2.20, 95%CI: 1.43–3.40). In patients with sepsis, ePVS was significantly associated with mortality (HR = 1.29, 95%CI: 1.14–1.46), and those with ePVS levels ≥5.79 had a significantly higher risk of death (HR = 2.91, 95%CI: 1.36–6.25). Finally, in non-heart failure patients, ePVS was significantly associated with 30-day mortality (HR = 1.31, 95%CI: 1.22–1.42), and those with ePVS levels ≥5.79 had a significantly higher risk of death (HR = 2.27, 95%CI: 1.50–3.45).

In Table S7 (HIS), the positive association between ePVS and 1-year mortality was also observed in several subgroups. In patients aged ≥60 years, ePVS was significantly associated with 1-year mortality (HR = 1.32, 95%CI: 1.17–1.48), with those having ePVS levels ≥5.79 showing a significantly increased risk of death (HR = 2.40, 95%CI: 1.65–3.49). In patients with a GCS score ≥ 13, ePVS was significantly associated with 1-year mortality (HR = 1.35, 95%CI: 1.16–1.58), and those with ePVS levels ≥5.79 had a significantly increased risk of death (HR = 4.57, 95%CI: 2.73–7.65). In patients with a SOFA score < 1, ePVS was significantly associated with 1-year mortality (HR = 1.34, 95%CI: 1.15–1.58), and those with ePVS levels ≥5.79 showed a significantly higher risk of death (HR = 2.63, 95%CI: 1.63–4.25). In non-AKI patients, ePVS was significantly associated with 1-year mortality (HR = 1.27, 95%CI: 1.18–1.36), and those with ePVS levels ≥5.79 showed a significantly higher risk of death (HR = 2.82, 95%CI: 2.05–3.88). Similarly, in patients with sepsis, ePVS was significantly associated with 1-year mortality (HR = 1.21, 95%CI: 1.10–1.33), and those with ePVS levels ≥5.79 had a significantly increased risk of death (HR = 2.82, 95%CI: 1.59–4.99). Lastly, in non-heart failure patients, ePVS was significantly associated with 1-year mortality (HR = 1.26, 95%CI: 1.17–1.35), and those with ePVS levels ≥5.79 had a significantly higher risk of death (HR = 2.73, 95%CI: 2.00–3.73).

### 3.4 Sensitivity analysis

Moreover, we assessed the association of ePVS calculated using another method (KH-ePVS) with 30-day and 1-year mortality in patients with ICH. As shown in Table 3, after adjusting for selected covariates, KH-ePVS was associated with an increased risk of both 30-day and 1-year mortality in ICH patients. For the MIMIC-IV data, KH-ePVS was not significantly associated with 30-day mortality (HR = 1.00, 95%CI: 0.91–1.10). However, patients with KH-ePVS levels ≥1.68 had a higher risk of 30-day mortality (HR = 1.35, 95%CI: 1.08–1.69). In the HIS dataset, KH-ePVS was significantly associated with 30-day mortality (HR = 1.04, 95%CI: 1.02–1.06). Patients with KH-ePVS levels ≥1.68 had a moderately higher risk of 30-day mortality, with an HR (95%CI) of 1.33 (0.91–1.96), although this result was not statistically significant (*p* = 0.144). In contrast, patients with KH-ePVS levels < −8.08 had a significantly lower risk of 30-day mortality (HR = 0.54, 95%CI: 0.33–0.87, *p* = 0.011).

**Table 3:** Association between KH-ePVS and 30-day and 1-year mortality in ICH patients.

Variables	Model 1		Model 2	
	HR (95% CI)	*P*	HR (95% CI)	*P*
30-day mortality				
MIMIC IV				
KH-ePVS	1.02 (0.95–1.08)	0.646	1.00 (0.91–1.10)	0.971
KH-ePVS levels				
[−8.08 to 1.68)	Ref		Ref	
< −8.08	0.89 (0.70–1.13)	0.333	1.06 (0.83–1.37)	0.624
≥ 1.68	1.54 (1.24–1.91)	**<0.001**	1.35 (1.08–1.69)	**0.007**
HIS				
KH-ePVS	1.04 (1.02–1.06)	**<0.001**	1.04 (1.03–1.06)	**<0.001**
KH-ePVS levels				
[−8.08 to 1.68)	Ref		Ref	
< −8.08	0.52 (0.33–0.84)	**0.008**	0.54 (0.33–0.87)	**0.011**
≥ 1.68	1.43 (0.98–2.08)	0.065	1.33 (0.91–1.96)	0.144
1-year mortality				
MIMIC IV				
KH-ePVS	1.02 (1.01–1.03)	**<0.001**	1.01 (1.01–1.12)	**<0.001**
KH-ePVS levels				
[−8.08 to 1.68)	Ref		Ref	
< −8.08	0.81 (0.68–0.97)	**0.019**	0.89 (0.74–1.07)	0.209
≥ 1.68	1.37 (1.17–1.62)	**<0.001**	1.28 (1.08–1.51)	**0.004**
HIS				
KH-ePVS	1.02 (1.01–1.03)	**0.001**	1.02 (1.01–1.03)	**0.001**
KH-ePVS levels				
[−8.08 to 1.68)	Ref		Ref	
< −8.08	0.75 (0.56–1.00)	**0.046**	0.76 (0.57–1.01)	0.058
≥ 1.68	1.33 (1.01–1.75)	**0.040**	1.33 (1.01–1.75)	**0.041**

The bold *P* values represented statistically significant. MIMIC-IV: Medical Information Mart for Intensive Care IV, HIS: Hospital Information System, ePVS: estimated plasma volume status, ICH: intracerebral hemorrhage, HR: hazard ratio, CI: confidence interval, Ref: reference. For 30-day mortality, Model 1: unadjusted model; Model 2: adjusted for age, ICU types, AG, race (only MIMIC-IV), vasopressors, mannitol, SOFA, CCI, glucose, β-blockers, mechanical ventilation and urine output. For 1-year mortality, Model 1: unadjusted model; Model 2: adjusted for age, race (only MIMIC-IV), ICU types, vasopressors, SOFA, CCI, platelet, glucose, and mechanical ventilation.

For 1-year mortality, the results were similarly significant. In the MIMIC-IV dataset, KH-ePVS was significantly associated with 1-year mortality (HR = 1.01, 95%CI: 1.01–1.12). Patients with KH-ePVS levels ≥1.68 had an HR (95%CI) of 1.28 (1.08–1.51). In the HIS dataset, KH-ePVS remained significantly associated with 1-year mortality (HR = 1.02, 95%CI: 1.01–1.03). Patients with KH-ePVS levels ≥1.68 had an HR (95%CI) of 1.33 (1.01–1.75), showing a significantly greater likelihood of mortality within one year.

### 3.5 Associations between ePVS levels and 30-day and 1-year mortality in RCS plots

**Figure 3:**
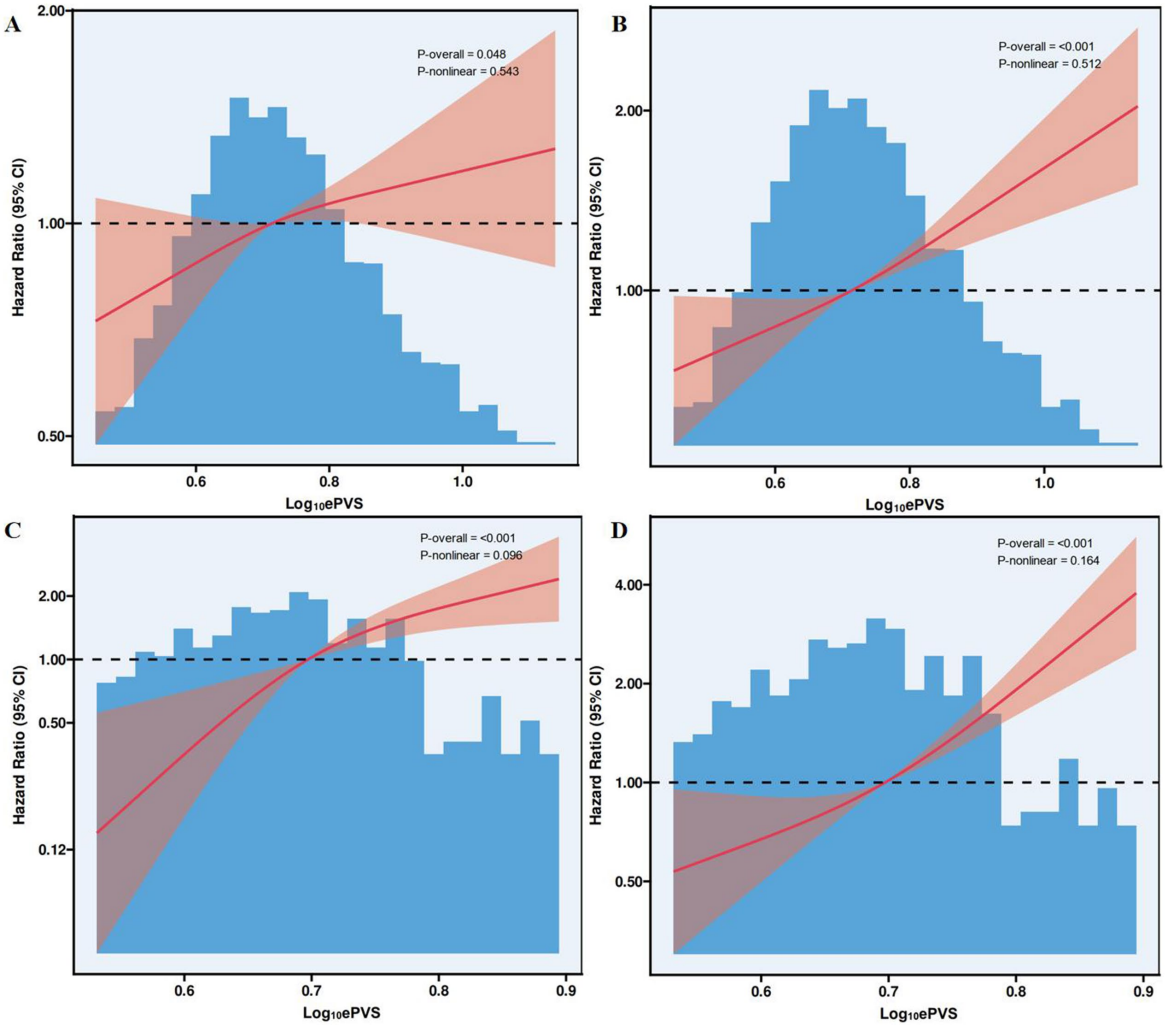
Association between ePVS levels and 30-day and 1-year mortality in the restricted cubic spline plots. **(A)** MIMIC-IV (30-day mortality); **(B)** MIMIC-IV (1-year mortality); **(C)** HIS (30-day mortality); **(D)** HIS (1-year mortality).

Based on the results from Figure 3, the RCS analysis showed a linear association between ePVS (i.e., Log-transformed PVS) and 30-day and 1-year mortality rates. Figure 3A demonstrated a statistically significant overall relationship between ePVS and 30-day mortality in the MIMIC-IV database (*p* = 0.048), while the nonlinear relationship was not significant (*p* = 0.543). Figure 3B indicated a significant linear relationship between ePVS and 1-year mortality in the MIMIC-IV database (*p* < 0.001), with no significant nonlinear relationship (*p* = 0.512). Figure 3C further analyzed the nonlinear association between ePVS and 30-day mortality in the HIS database. Although the overall *p*-value was less than 0.001, the nonlinear *p*-value was 0.096, suggesting weaker evidence for a nonlinear association. Finally, Figure 3D showed a *p*-value of 0.164 for the nonlinear relationship between ePVS and 1-year mortality in the HIS database, further supporting the dominance of the linear relationship.

## 4 Discussion

This study investigated the association between ePVS and 30-day and 1-year mortality in patients with ICH. These results suggest that elevated ePVS is associated with an increased risk of 30-day and 1-year mortality. Compared to patients with ePVS levels of [4.63–5.79), those with ePVS levels of ≥5.79 had a higher risk of 30-day and 1-year mortality.

To the best of our knowledge, no study has discussed the role of PVS in the prognosis of patients with ICH. As mentioned previously, ePVS is calculated using Hb and HCT, which are common indices in the ICU, and is a low-cost, rapid quantification method for monitoring plasma volume fluctuations. According to previous studies, ePVS is a potential prognostic factor in multiple diseases. Gao et al. (12) explored the association between ePVS and the 28-day mortality rate in patients with septic shock admitted to the ICU based on the MIMIC-IV database and showed a J-shaped association between ePVS and 28-day mortality, with higher ePVS levels associated with an increased risk of mortality. Another single-center prospective observational study that included 100 ICU patients with sepsis or septic shock suggested that ePVS was correlated with the amount of intravenous fluid resuscitation and may be used as a simple and novel prognostic factor in patients with sepsis or septic shock (16). Niedermeyer et al. (17) conducted a retrospective cohort study of patients with acute respiratory distress syndrome (ARDS) and observed that PVS was independently linked to mortality, ICU-free days, and ventilator-free days in patients with ARDS, which could be considered for risk stratification and direct therapy, particularly fluid management. In our study, we similarly extracted data from the MIMIC-IV database and the HIS and evaluated the association between ePVS and 30-day and 1-year mortality in patients with ICH, finding that the highest level of ePVS (≥5.79) was associated with an increased risk of 30-day and 1-year mortality. Our findings partially addressed the gaps in the literature, indicating that timely monitoring and maintenance of ePVS at low levels in ICH patients may help reduce the potential risk of mortality in clinical settings. Nevertheless, since this retrospective study was not able to conclude a causal association between PVS and mortality risk in patients with ICH, future studies could focus on this aspect, as well as explore the optimal range of PVS in this population. Our study confirms that elevated ePVS is independently associated with increased 30-day mortality in ICH patients. However, when compared to other well-established risk factors such as SOFA score, mechanical ventilation, and vasopressor use, the relative impact of ePVS appears to be moderate. While ePVS remains a statistically significant predictor, its HR is lower than that of SOFA and mechanical ventilation. This suggests that plasma volume status contributes to mortality risk assessment but may not replace conventional severity scores in ICU settings. Instead, ePVS could serve as an early warning marker for hemodynamic instability, which may be particularly valuable in guiding fluid management in ICH patients. Future studies should explore the integration of ePVS into existing ICU severity scores (e.g., SOFA and APACHE) to determine whether it provides additional prognostic value.

The mechanisms by which elevated ePVS are linked to an increased risk of 30-day and 1-year mortality in patients with ICH may be complex and varied. Increased plasma volume may contribute to hemodilution, leading to decreased HCT and reduced coagulation factor concentrations, which could exacerbate hemorrhagic progression in ICH patients. Hemodilution has been associated with coagulation dysfunction, impaired platelet aggregation, and prolonged PT and activated partial thromboplastin time (APTT), all of which may increase the risk of hematoma expansion and worsen patient outcomes. Low Hb levels are associated with poor prognosis in ICH patients (18). Roh et al. (19) found that a lower Hb level on ICU admission was related to greater hematoma expansion after ICH, which was associated with worse outcomes. Anemia may lead to cellular energy dysfunction, neuronal tissue hypoxia, and metabolic distress, which can cause pronounced secondary cerebral injury by reducing the oxygen-carrying capacity (20). In samples from the MIMIC-IV database, the incidence of anemia was 60.32%, and packed red blood cell (RBC) transfusion may be considered for patients with anemia (21). However, our study found no significant association between RBC transfusion and 30-day mortality, suggesting that the detrimental effects of hemodilution and increased plasma volume may not be effectively counteracted by transfusion alone. In the present study, transfusion situations among the three ePVS level groups were significantly different; however, no association between transfusion and 30-day and 1-year mortality was observed, indicating that, regardless of whether ICH patients underwent RBC transfusion, a higher ePVS level was linked to an increased risk of 30-day and 1-year mortality. Moreover, hematoma in ICH can trigger an inflammatory response, which promotes post-ICH brain injury and results in a poorer prognosis (22). In addition to ePVS, we assessed the association between KH-ePVS (calculated based on HCT and weight) and 30-day and 1-year mortality in patients with ICH and obtained similar results. This further suggests that the association we found is relatively credible.

Moreover, the relationship between ePVS and mortality in patients with ICH was evaluated in different subgroups. Among ICH patients with non-HF, non-sepsis, AKI, non-AKI, SOFA ≥1, GCS ≥13, age < 60, or age ≥ 60, a higher level of ePVS was associated with an increased risk of 30-day and 1-year mortality. One study showed that age > 65 years is an important predictor of 30-day mortality in a subgroup of ICH patients (23). According to our results, in both older and younger patients, it is important to focus on ePVS levels at ICU admission. Pana et al. (24) found that HF increases the risk of mortality in both acute ischemic stroke (AIS) and ICH. HF is a recognized risk factor for stroke, and the proposed mechanisms include thromboembolism, small vessel endothelial damage, and cerebral hypoperfusion (25). In our subgroup analysis, the association between ePVS and 30-day mortality was only significant in patients with ICH without HF. Although the cause of this phenomenon is unknown, clinicians’ targeted treatment of patients with HF may be involved. In addition, we found that an elevated ePVS level was associated with an increased risk of 30-day and 1-year mortality, regardless of whether patients with ICH had AKI. However, a meta-analysis provided evidence that AKI is a common complication following both AIS and ICH and is associated with increased mortality following AIS, but not ICH (26). Moreover, the SOFA score and GCS are common indices for evaluating the severity of the disease in the ICU, and our research suggests that among the study subjects who had a worse situation, ePVS had a positive association with an increased risk of 30-day and 1-year mortality.

The study was based on the MIMIC-IV database, which contains a large real patient sample, making the findings reliable to some extent. By investigating the association between ePVS levels and 30-day and 1-year mortality in patients with ICH, the study provides a reference for risk stratification and prognostic improvement in this population. Additionally, the results of the subgroup analysis may provide a basis for population applicability. Various potential confounding factors in the ICU setting that may have influenced the results, including comorbidities, laboratory indicators, and treatment, were also included. Nevertheless, some limitations exist in the conclusions. However, several limitations should be acknowledged. Due to the retrospective cohort study design, selection bias is unavoidable. Some important clinical variables, such as the cause of ICH, hematoma location and volume, midline shift, and stroke severity scores like NIHSS and APACHE, were not available in the database, limiting the adjustment for more comprehensive confounders. Additionally, while we included serum creatinine as a renal function marker, eGFR was not directly available, which may have led to incomplete adjustment for renal impairment. Certain in-hospital complications, such as pneumonia, deep vein thrombosis, and rebleeding, were not systematically recorded, though major complications like AKI, sepsis, and heart failure, which are known to significantly impact mortality, were accounted for. Moreover, detailed neurosurgical data, including hematoma evacuation and surgical interventions, were lacking, which may have influenced mortality outcomes.

Another key limitation is that ePVS was calculated using hematocrit and hemoglobin levels measured within the first 24 h of ICU admission, meaning it does not reflect dynamic changes over time. Fluid shifts, blood loss, and hemodynamic fluctuations are common in critically ill patients, and serial ePVS measurements could provide additional prognostic value. Furthermore, because our study exclusively included ICU inpatients, the applicability of ePVS to non-ICU patients remains uncertain. ICU patients generally have more severe ICH, higher complication rates, and receive more intensive medical interventions, meaning the findings may not be directly generalizable to patients with milder ICH who do not require ICU admission. Future studies should validate these findings in broader populations, and investigate the potential role of serial ePVS measurements in guiding fluid management and risk assessment in ICH patients.

Due to the retrospective cohort study design, selection bias was difficult to avoid. Information on characteristics such as the cause of ICH and CT features of hemorrhagic areas was not available in the database, which limited the adjustment of more comprehensive confounding factors. Additionally, because participants from the MIMIC database have limited representation, the applicability of ePVS in the mortality of patients requires further exploration.

## 5 Conclusion

A higher ePVS level was associated with an increased risk of 30-day and 1-year mortality in ICU patients with ICH, suggesting that timely monitoring of ePVS levels and adjustment of the therapeutic schedule may be beneficial for reducing the potential mortality risk in this population. Furthermore, the causal association and potential mechanisms underlying the prognoses of PVS and ICH need to be clarified.

## Data Availability

Publicly available datasets were analyzed in this study. This data can be found here: MIMIC-IV wabpage: https://mimic.mit.edu/docs/iv/.

## Footnotes

^1^ https://mimic.mit.edu/docs/iv/
