# Supplementary material for: Associations of estimated plasma volume status with 30-day mortality and 1-year mortality in patients with intracerebral hemorrhage: a study of the MIMIC-IV database and the hospital information system
Source: Front Neurol. 2025 Apr 1;16:1548064. doi: 10.3389/fneur.2025.1548064 (PMC11996633; doi:10.3389/fneur.2025.1548064)
Supplement: Supplementary file 1 [file Supplementary_file_1.docx]

Table S1. Variables with missing data

| Variables | N (%) |
| --- | --- |
| HR | 5 (0.20) |
| RR | 100 (3.98) |
| Temperature | 9 (0.36) |
| SpO_2_ | 4 (0.16) |
| WBC | 1 (0.04) |
| Platelet | 3 (0.12) |
| Cr | 7 (0.28) |
| BUN | 8 (0.32) |
| Glucose | 8 (0.32) |
| Ca | 98 (3.90) |
| Na | 4 (0.16) |
| K | 4 (0.16) |
| Chloride | 6 (0.24) |
| Bicarbonate | 7 (0.28) |
| PT | 238 (9.47) |
| SOFA | 3 (0.12) |
| GCS | 3 (0.12) |
| Urine output | 82 (3.26) |
| MAP | 3 (0.12) |
| AG | 8 (0.32) |

HR: heart rate, RR: respiratory rate, WBC: white blood cell, Cr: creatinine, BUN: blood urea nitrogen, Ca: calcium, Na: sodium, K: potassium, PT: prothrombin time, SOFA: the Sequential Organ-Failure Assessment, GCS: the Glasgow Coma Scale, MAP: mean arterial pressure, AG: anion gap.

Table S2. Characteristics of patients before and after the multiple interpolation of missing data

| Variables | After interpolation  (n=2512) | Before interpolation  (n=2512) | Statistics | *P* |
| --- | --- | --- | --- | --- |
| HR, bpm, Mean ± SD | 82.87 ± 17.64 | 82.84 ± 17.63 | t=0.06 | 0.951 |
| RR, insp/min, Mean ± SD | 18.64 ± 5.23 | 18.65 ± 5.19 | t=-0.05 | 0.958 |
| Temperature, ℃, Mean ± SD | 36.84 ± 0.65 | 36.84 ± 0.65 | t=-0.10 | 0.921 |
| SpO_2_, %, Mean ± SD | 97.38 ± 3.31 | 97.38 ± 3.31 | t=-0.03 | 0.974 |
| WBC, K/uL, M (Q_1_, Q_3_) | 10.25 (7.90, 13.20) | 10.30 (7.90, 13.20) | Z=0.022 | 0.982 |
| Platelet, K/uL, M (Q_1_, Q_3_) | 206.00 (161.00, 259.00) | 206.00 (161.00, 259.00) | Z=-0.009 | 0.993 |
| Cr, mg/dL, M (Q_1_, Q_3_) | 0.90 (0.70, 1.10) | 0.90 (0.70, 1.10) | Z=-0.082 | 0.934 |
| BUN, mg/dL, M (Q_1_, Q_3_) | 16.00 (12.00, 22.00) | 16.00 (12.00, 22.00) | Z=0.013 | 0.990 |
| Glucose, mg/dL, M (Q_1_, Q_3_) | 129.00 (107.00, 160.00) | 129.00 (107.00, 160.00) | Z=0.054 | 0.957 |
| Ca, mg/dL, Mean ± SD | 8.73 ± 0.73 | 8.74 ± 0.73 | t=-0.04 | 0.969 |
| AG, Mean ± SD | 16.31 ± 3.91 | 16.32 ± 3.91 | t=-0.02 | 0.984 |
| PT, sec, M (Q_1_, Q_3_) | 12.60 (11.80, 14.00) | 12.60 (11.70, 14.00) | Z=-0.135 | 0.893 |
| SOFA, M (Q_1_, Q_3_) | 1.00 (0.00, 2.00) | 1.00 (0.00, 2.00) | Z=-0.006 | 0.995 |
| GCS, Mean ± SD | 13.99 ± 2.04 | 13.99 ± 2.04 | t=0.01 | 0.989 |
| Urine output, ml, M (Q_1_, Q_3_) | 1550.00 (990.00, 2278.00) | 1555.00 (995.00, 2285.00) | Z=0.220 | 0.826 |
| MAP, mmHg, Mean ± SD | 96.23 ± 17.18 | 96.23 ± 17.19 | t=-0.01 | 0.990 |

HR: heart rate, SD: standard deviation, RR: respiratory rate, WBC: white blood cell, M: median, Q_1_: 1st quartile, Q_3_: 3rd quartile, Cr: creatinine, BUN: blood urea nitrogen, Ca: calcium, AG: anion gap, PT: prothrombin time, SOFA: the Sequential Organ-Failure Assessment, GCS: the Glasgow Coma Scale, MAP: mean arterial pressure.

Table S3. Characteristics of ICH patients in different ePVS level groups

| Variables | Total  (n=515) | ePVS levels | | | Statistics | *P* |
| --- | --- | --- | --- | --- | --- | --- |
|  |  | <4.63  (n=220) | [4.63-5.79)  (n=192) | ≥5.79  (n=103) |  |  |
| Age, years, Mean ± SD | 61.67 ± 11.41 | 61.26 ± 11.43 | 62.13 ± 10.87 | 62.13 ± 10.87 | F=0.298 | 0.742 |
| Gender, n (%) |  |  |  |  | χ^2^=3.900 | 0.142 |
| Female | 331 (64.27) | 152 (69.09) | 117 (60.94) | 62 (60.19) |  |  |
| Male | 184 (35.73) | 68 (30.91) | 75 (39.06) | 41 (39.81) |  |  |
| ICU types, n (%) |  |  |  |  | χ^2^=8.381 | 0.079 |
| Neuro intermediate/stepdown | 195 (37.86) | 72 (32.73) | 83 (43.23) | 40 (38.83) |  |  |
| SICU | 288 (55.92) | 138 (62.73) | 94 (48.96) | 56 (54.37) |  |  |
| Others | 199 (7.92) | 43 (5.19) | 43 (5.04) | 113 (13.61) |  |  |
| Cerebral infarction, n (%) |  |  |  |  | χ^2^=0.863 | 0.650 |
| No | 391 (75.92) | 163 (74.09) | 147 (76.56) | 81 (78.64) |  |  |
| Yes | 124 (24.08) | 57 (25.91) | 45 (23.44) | 22 (21.36) |  |  |
| DM, n (%) |  |  |  |  | χ^2^=1.408 | 0.495 |
| No | 405 (78.64) | 171 (77.73) | 156 (81.25) | 78 (75.73) |  |  |
| Yes | 110 (21.36) | 49 (22.27) | 36 (18.75) | 25 (24.27) |  |  |
| HF, n (%) |  |  |  |  | χ^2^=0.146 | 0.930 |
| No | 483 (93.79) | 206 (93.64) | 181 (94.27) | 96 (93.20) |  |  |
| Yes | 32 (6.21) | 14 (6.36) | 11 (5.73) | 7 (6.80) |  |  |
| AKI, n (%) |  |  |  |  | χ^2^=3.554 | 0.169 |
| No | 471 (91.46) | 206 (93.64) | 175 (91.15) | 90 (87.38) |  |  |
| Yes | 44 (8.54) | 14 (6.36) | 17 (8.85) | 13 (12.62) |  |  |
| Sepsis, n (%) |  |  |  |  | χ^2^=1.602 | 0.449 |
| No | 378 (73.40) | 158 (71.82) | 147 (76.56) | 73 (70.87) |  |  |
| Yes | 137 (26.60) | 62 (28.18) | 45 (23.44) | 30 (29.13) |  |  |
| HR, bpm, Mean ± SD | 84.92 ± 19.35 | 85.35 ± 21.16 | 83.63 ± 18.32 | 86.38 ± 17.09 | F=0.774 | 0.462 |
| MAP, mmHg, Mean ± SD | 97.38 ± 17.19 | 97.67 ± 17.71 | 97.49 ± 17.05 | 96.53 ± 16.44 | F=0.162 | 0.850 |
| RR, insp/min, Mean ± SD | 18.97 ± 5.56 | 19.23 ± 5.45 | 19.02 ± 5.58 | 18.34 ± 5.76 | F=0.905 | 0.405 |
| Temperature, ℃, Mean ± SD | 36.83 ± 0.65 | 36.78 ± 0.70 | 36.83 ± 0.58 | 36.93 ± 0.65 | F=1.781 | 0.189 |
| SpO_2_, %, Mean ± SD | 97.29 ± 4.08 | 97.46 ± 2.83 | 97.22 ± 5.40 | 97.05 ± 3.47 | F=0.394 | 0.675 |
| SOFA, M (Q_1_, Q_3_) | 1.00 (0.00, 2.00) | 1.00 (0.00, 3.00) | 1.00 (0.00, 2.00) | 1.00 (0.00, 3.00) | W=6.290 | 0.043 |
| GCS, Mean ± SD | 11.29 ± 4.13 | 11.85 ± 3.89 | 11.33 ± 4.03 | 9.98 ± 4.55 | F=7.408 | **<0.001** |
| CCI, M (Q_1_, Q_3_) | 3.00 (2.00, 5.00) | 3.00 (1.00, 5.00) | 3.00 (2.00, 5.00) | 4.00 (2.00, 5.00) | W=2.899 | 0.235 |
| WBC, K/uL, M (Q_1_, Q_3_) | 9.91 (7.19, 13.34) | 9.29 (6.70,12.77) | 9.76 (7.37,13.11) | 10.94 (7.62,15.34) | W=7.362 | **0.025** |
| Platelet, K/uL, M (Q_1_, Q_3_) | 209.00 (168.00, 260.00) | 210.50 (172.00,257.25) | 207.00 (166.00,259.25) | 209.00 (159.50,271.50) | W=0.215 | 0.898 |
| Cr, mg/dL, M (Q_1_, Q_3_) | 0.90 (0.70, 1.10) | 0.90 (0.70,1.10) | 0.90 (0.70, 1.10) | 0.90 (0.70, 1.20) | W=2.324 | 0.313 |
| BUN, mg/dL, M (Q_1_, Q_3_) | 6.20 (4.89, 8.20) | 6.00 (4.79,7.88) | 6.24 (4.83,8.10) | 6.61 (5.52,9.48) | W=7.107 | **0.029** |
| Glucose, mg/dL, M (Q_1_, Q_3_) | 127.00 (107.00, 149.50) | 126.50 (107.75, 147.00) | 126.00 (107.00, 155.50) | 128.00 (107.00, 149.50) | W=0.195 | 0.907 |
| Ca, mg/dL, Mean ± SD | 8.73 ± 0.80 | 8.72 ± 0.86 | 8.76 ± 0.77 | 8.69 ± 0.72 | F=0.268 | 0.765 |
| AG, Mean ± SD | 16.29 ± 3.99 | 16.23 ± 4.59 | 16.03 ± 3.35 | 16.91 ± 3.68 | F=1.692 | 0.185 |
| PT, sec, M (Q_1_, Q_3_) | 11.90 (11.20, 12.95) | 12.00 (11.30,13.03) | 11.90 (11.10,12.83) | 11.60 (11.10,12.50) | W=4.296 | 0.117 |
| Urine output, ml, M (Q_1_, Q_3_) | 1550.00 (1000.00, 2252.50) | 1590.00 (972.50,2293.75) | 1497.50 (991.50,2161.25) | 1550.00 (1100.00,2237.50) | W=0.724 | 0.696 |
| Brain surgery, n (%) |  |  |  |  | χ^2^=1.934 | 0.380 |
| No | 498 (96.70) | 215 (97.73) | 183 (95.31) | 100 (97.09) |  |  |
| Yes | 17 (3.30) | 5 (2.27) | 9 (4.69) | 3 (2.91) |  |  |
| RRT, n (%) |  |  |  |  | χ^2^=0.753 | 0.686 |
| No | 461 (89.51) | 197 (89.55) | 174 (90.62) | 90 (87.38) |  |  |
| Yes | 54 (10.49) | 23 (10.45) | 18 (9.38) | 13 (12.62) |  |  |
| Mechanical ventilation, n (%) |  |  |  |  | χ^2^=5.007 | 0.082 |
| No | 182 (35.34) | 71 (32.27) | 65 (33.85) | 46 (44.66) |  |  |
| Yes | 333 (64.66) | 149 (67.73) | 127 (66.15) | 57 (55.34) |  |  |
| Vasopressors, n (%) |  |  |  |  | χ^2^=0.766 | 0.682 |
| No | 410 (79.61) | 179 (81.36) | 151 (78.65) | 80 (77.67) |  |  |
| Yes | 105 (20.39) | 41 (18.64) | 41 (21.35) | 23 (22.33) |  |  |
| Mannitol, n (%) |  |  |  |  | χ^2^=0.844 | 0.656 |
| No | 469 (91.07) | 202 (91.82) | 172 (89.58) | 95 (92.23) |  |  |
| Yes | 46 (8.93) | 18 (8.18) | 20 (10.42) | 8 (7.77) |  |  |
| Furosemide, n (%) |  |  |  |  | χ^2^=6.288 | **0.043** |
| No | 400 (77.67) | 179 (81.36) | 150 (78.12) | 71 (68.93) |  |  |
| Yes | 115 (22.33) | 41 (18.64) | 42 (21.88) | 32 (31.07) |  |  |
| Transfusion, n (%) |  |  |  |  | χ^2^=10.023 | 0.311 |
| Non-transfusion | 454 (88.16) | 198 (90.00) | 166 (86.46) | 90 (87.38) |  |  |
| Plasma | 10 (1.94) | 2 (0.91) | 6 (3.12) | 2 (1.94) |  |  |
| Thrombocyte | 21 (4.08) | 8 (3.64) | 6 (3.12) | 7 (6.80) |  |  |
| RBC | 18 (3.50) | 9 (4.09) | 6 (3.12) | 3 (2.91) |  |  |
| Others | 12 (2.33) | 3 (1.36) | 8 (4.17) | 1 (0.97) |  |  |
| β-blockers, n (%) |  |  |  |  | χ^2^=0.198 | 0.906 |
| No | 149 (28.93) | 65 (29.55) | 56 (29.17) | 28 (27.18) |  |  |
| Yes | 366 (71.07) | 155 (70.45) | 136 (70.83) | 75 (72.82) |  |  |
| 30-day mortality, n (%) |  |  |  |  | χ^2^=36.963 | **<0.001** |
| No | 383 (74.37) | 185 (84.09) | 144 (75.00) | 54 (52.43) |  |  |
| Yes | 132 (25.63) | 35 (15.91) | 48 (25.00) | 49 (47.57) |  |  |
| 1-year mortality, n (%) |  |  |  |  | χ^2^=41.725 | **<0.001** |
| No | 227 (44.10) | 105 (47.70) | 105 (54.70) | 17 (16.50) |  |  |
| Yes | 288 (55.90) | 115 (52.30) | 87 (45.30) | 86 (83.50) |  |  |
| Follow-up time, days, M (Q_1_, Q_3_) | 30.00 (26.61, 30.00) | 30.00 (30.00, 30.00) | 30.00 (29.57, 30.00) | 30.00 (8.5, 30.00) | W=38.235 | **<0.001** |

F: ANOVA, W: Kruskal-Wallis H test, χ^2^: chi-square test.

The bold P values represented statistically significant.

ICH: intracerebral hemorrhage, ePVS: estimated plasma volume status, SD: standard deviation, ICU: intensive care unit, SICU: surgical intensive care unit, DM: diabetes mellitus, HF: heart failure, AKI: acute kidney injury, HR: heart rate, MAP: mean arterial pressure, RR: respiratory rate, SOFA: the Sequential Organ-Failure Assessment, M: median, Q_1_: 1st quartile, Q_3_: 3rd quartile, GCS: the Glasgow Coma Scale, CCI: the Charlson Comorbidity Index, WBC: white blood cell, Cr: creatinine, BUN: blood urea nitrogen, Ca: calcium, AG: anion gap, PT: prothrombin time, RRT: renal replacement therapy, RBC: red blood cell.

Table S4. Association between ePVS and 30-day mortality in different subgroups for MIMIC-IV

| Variables | Age <60  (n=772) | | Age ≥60  (n=1740) | |
| --- | --- | --- | --- | --- |
|  | HR (95% CI) | *P* | HR (95% CI) | *P* |
| ePVS | 1.02 (0.95-1.09) | 0.559 | 1.05 (1.01-1.11) | **0.048** |
| ePVS levels |  |  |  |  |
| [4.63-5.79) | Ref |  | Ref |  |
| <4.63 | 0.99 (0.63-1.57) | 0.974 | 1.05 (0.83-1.33) | 0.700 |
| ≥5.79 | 1.80 (1.13-2.86) | **0.013** | 1.28 (1.04-1.58) | **0.021** |
| Variables | GCS <13  (n=348) | | GCS ≥13  (n=2164) | |
|  | HR (95% CI) | *P* | HR (95% CI) | *P* |
| ePVS | 1.09 (0.94-1.26) | 0.240 | 1.05 (1.01-1.09) | **0.025** |
| ePVS levels |  |  |  |  |
| [4.63-5.79) | Ref |  | Ref |  |
| <4.63 | 1.60 (0.96-2.66) | 0.073 | 0.90 (0.72-1.14) | 0.382 |
| ≥5.79 | 1.55 (0.95-2.53) | 0.081 | 1.35 (1.10-1.66) | **0.005** |
| Variables | SOFA <1  (n=1197) | | SOFA ≥1  (n=1315) | |
|  | HR (95% CI) | *P* | HR (95% CI) | *P* |
| ePVS | 1.12 (1.04-1.21) | **0.003** | 1.03 (0.98-1.08) | 0.209 |
| ePVS levels |  |  |  |  |
| [4.63-5.79) | Ref |  | Ref |  |
| <4.63 | 0.94 (0.68-1.30) | 0.711 | 1.08 (0.82-1.43) | 0.569 |
| ≥5.79 | 1.29 (0.95-1.74) | 0.098 | 1.39 (1.09-1.79) | **0.009** |
| Variables | Non-AKI  (n=915) | | AKI  (n=1597) | |
|  | HR (95% CI) | *P* | HR (95% CI) | *P* |
| ePVS | 1.14 (1.02-1.28) | **0.022** | 1.03 (0.99-1.07) | 0.213 |
| ePVS levels |  |  |  |  |
| [4.63-5.79) | Ref |  | Ref |  |
| <4.63 | 1.26 (0.83-1.90) | 0.282 | 0.93 (0.73-1.18) | 0.549 |
| ≥5.79 | 1.58 (1.11-2.24) | **0.011** | 1.26 (1.01-1.58) | **0.049** |
| Variables | Non-sepsis  (n=1836) | | Sepsis  (n=676) | |
|  | HR (95% CI) | *P* | HR (95% CI) | *P* |
| ePVS | 1.06 (1.01-1.11) | **0.021** | 1.03 (0.97-1.11) | 0.333 |
| ePVS levels |  |  |  |  |
| [4.63-5.79) | Ref |  | Ref |  |
| <4.63 | 1.06 (0.83-1.36) | 0.650 | 0.86 (0.59-1.26) | 0.449 |
| ≥5.79 | 1.42 (1.13-1.79) | **0.003** | 1.21 (0.85-1.72) | 0.282 |
| Variables | Non-HF  (n=2158) | | HF  (n=354) | |
|  | HR (95% CI) | *P* | HR (95% CI) | *P* |
| ePVS | 1.06 (1.01-1.10) | **0.015** | 1.06 (0.98-1.16) | 0.165 |
| ePVS levels |  |  |  |  |
| [4.63-5.79) | Ref |  | Ref |  |
| <4.63 | 1.03 (0.82-1.29) | 0.816 | 0.80 (0.46-1.37) | 0.411 |
| ≥5.79 | 1.46 (1.18-1.80) | **<0.001** | 0.99 (0.61-1.58) | 0.951 |

The bold numbers represented statistically significant.

ePVS: estimated plasma volume status, HR: hazard ratio, CI: confidence interval, Ref: reference, GCS: the Glasgow Coma Scale, SOFA: the Sequential Organ-Failure Assessment, AKI: acute kidney injury, HF: heart failure.

Model 1: unadjusted model;

Model 2 for age subgroups: adjusted for race, ICU types, HR, SpO_2_, SOFA, CCI, platelet, glucose, AG, urine output, mechanical ventilation, vasopressors, mannitol and β-blockers;

Model 2 for SOFA subgroups: adjusted for age, race, ICU types, HR, SpO_2_, CCI, platelet, glucose, AG, urine output, mechanical ventilation, vasopressors, mannitol and β-blockers;

Model 2 for GCS, AKI, sepsis and HF subgroups: adjusted for age, race, ICU types, HR, SpO_2_, SOFA, CCI, platelet, glucose, AG, urine output, mechanical ventilation, vasopressors, mannitol and β-blockers.

Table S5. Association between ePVS and 1-year mortality in different subgroups for MIMIC-IV

| Variables | Age <60  (n=772) | | Age ≥60  (n=1740) | |
| --- | --- | --- | --- | --- |
|  | HR (95% CI) | *P* | HR (95% CI) | *P* |
| ePVS | 1.07 (1.02-1.11) | **0.005** | 1.11 (1.07-1.15) | **<0.001** |
| ePVS levels |  |  |  |  |
| [4.63-5.79) | Ref |  | Ref |  |
| <4.63 | 0.62 (0.47-0.83) | **0.001** | 0.73 (0.61-0.86) | **<0.001** |
| ≥5.79 | 1.15 (0.87-1.51) | 0.323 | 1.25 (1.08-1.44) | **0.003** |
| Variables | GCS <13  (n=348) | | GCS ≥13  (n=2164) | |
|  | HR (95% CI) | *P* | HR (95% CI) | *P* |
| ePVS | 1.12 (1.01-1.24) | **0.035** | 1.05 (1.01-1.09) | **0.025** |
| ePVS levels |  |  |  |  |
| [4.63-5.79) | Ref |  | Ref |  |
| <4.63 | 0.73 (0.49-1.09) | 0.120 | 0.70 (0.60-0.82) | **<0.001** |
| ≥5.79 | 1.25 (0.89-1.76) | 0.203 | 1.25 (1.08-1.44) | **0.002** |
| Variables | SOFA <1  (n=1197) | | SOFA ≥1  (n=1315) | |
|  | HR (95% CI) | *P* | HR (95% CI) | *P* |
| ePVS | 1.13 (1.08-1.19) | **<0.001** | 1.08 (1.04-1.12) | **<0.001** |
| ePVS levels |  |  |  |  |
| [4.63-5.79) | Ref |  | Ref |  |
| <4.63 | 0.67 (0.54-0.83) | **<0.001** | 0.77 (0.63-0.95) | **0.013** |
| ≥5.79 | 1.27 (1.04-1.54) | **0.020** | 1.26 (1.06-1.50) | **0.008** |
| Variables | Non-AKI  (n=915) | | AKI  (n=1597) | |
|  | HR (95% CI) | *P* | HR (95% CI) | *P* |
| ePVS | 1.15 (1.07-1.23) | **<0.001** | 1.09 (1.06-1.12) | <0.001 |
| ePVS levels |  |  |  |  |
| [4.63-5.79) | Ref |  | Ref |  |
| <4.63 | 0.67 (0.51-0.87) | **0.003** | 0.74 (0.62-0.89) | **0.001** |
| ≥5.79 | 1.36 (1.09-1.70) | **0.007** | 1.20 (1.02-1.41) | **0.025** |
| Variables | Non-sepsis  (n=1836) | | Sepsis  (n=676) | |
|  | HR (95% CI) | *P* | HR (95% CI) | *P* |
| ePVS | 1.11 (1.08-1.15) | **<0.001** | 1.08 (1.02-1.13) | **0.004** |
| ePVS levels |  |  |  |  |
| [4.63-5.79) | Ref |  | Ref |  |
| <4.63 | 0.68 (0.57-0.81) | **<0.001** | 0.73 (0.54-0.98) | **0.034** |
| ≥5.79 | 1.24 (1.06-1.45) | **0.006** | 1.30 (1.00-1.70) | 0.053 |
| Variables | Non-HF  (n=2158) | | HF  (n=354) | |
|  | HR (95% CI) | *P* | HR (95% CI) | *P* |
| ePVS | 1.10 (1.07-1.14) | **<0.001** | 1.09 (1.03-1.16) | **0.002** |
| ePVS levels |  |  |  |  |
| [4.63-5.79) | Ref |  | Ref |  |
| <4.63 | 0.70 (0.60-0.82) | **<0.001** | 0.73 (0.49-1.09) | 0.118 |
| ≥5.79 | 1.31 (1.14-1.51) | **<0.001** | 0.94 (0.67-1.33) | 0.736 |

The bold numbers represented statistically significant.

ePVS: estimated plasma volume status, HR: hazard ratio, CI: confidence interval, Ref: reference, GCS: the Glasgow Coma Scale, SOFA: the Sequential Organ-Failure Assessment, AKI: acute kidney injury, HF: heart failure.

The subgroup analysis adjusted for age, race, ICU types, HR, SpO_2_, SOFA, CCI, platelet, glucose, AG, brain surgery, mechanical ventilation, vasopressors, mannitol and transfusion, but excluded subgroup variables.

Table S6. Association between ePVS and 30-day mortality in different subgroups for HIS

| Variables | Age <60  (n=200) | | Age ≥60  (n=315) | |
| --- | --- | --- | --- | --- |
|  | HR (95% CI) | *P* | HR (95% CI) | *P* |
| ePVS | 1.28 (1.15-1.42) | **<0.001** | 1.55 (1.35-1.79) | **<0.001** |
| ePVS levels |  |  |  |  |
| [4.63-5.79) | Ref |  | Ref |  |
| <4.63 | 0.67 (0.29-1.54) | 0.348 | 0.59 (0.35-0.99) | **0.045** |
| ≥5.79 | 2.72 (1.27-5.81) | **0.010** | 2.25 (1.40-3.63) | **0.001** |
| Variables | GCS <13  (n=221) | | GCS ≥13  (n=294) | |
|  | HR (95% CI) | *P* | HR (95% CI) | *P* |
| ePVS | 1.22 (1.13-1.31) | **<0.001** | 1.05 (1.01-1.09) | **0.025** |
| ePVS levels |  |  |  |  |
| [4.63-5.79) | Ref |  | Ref |  |
| <4.63 | 0.69 (0.43-1.10) | 0.123 | 0.31 (0.08-1.20) | 0.090 |
| ≥5.79 | 2.23 (1.44-3.45) | **<0.001** | 1.77 (0.55-5.67) | 0.340 |
| Variables | SOFA <1  (n=205) | | SOFA ≥1  (n=310) | |
|  | HR (95% CI) | *P* | HR (95% CI) | *P* |
| ePVS | 1.56 (1.28-1.91) | **<0.001** | 1.30 (1.19-1.41) | <0.001 |
| ePVS levels |  |  |  |  |
| [4.63-5.79) | Ref |  | Ref |  |
| <4.63 | 0.48 (0.24-0.95) | **0.035** | 0.80 (0.44-1.43) | 0.447 |
| ≥5.79 | 1.90 (1.01-3.56) | **0.046** | 2.76 (1.61-4.75) | **<0.001** |
| Variables | Non-AKI  (n=471) | | AKI  (n=44) | |
|  | HR (95% CI) | *P* | HR (95% CI) | *P* |
| ePVS | 1.14 (1.02-1.28) | **0.022** | 1.74 (1.21-2.50) | **0.003** |
| ePVS levels |  |  |  |  |
| [4.63-5.79) | Ref |  | Ref |  |
| <4.63 | 1.34 (1.24-1.45) | **<0.001** | 0.51 (0.08-3.24) | 0.474 |
| ≥5.79 | 2.20 (1.43-3.40) | **<0.001** | 3.47 (0.71-17.08) | 0.126 |
| Variables | Non-sepsis  (n=378) | | Sepsis  (n=137) | |
|  | HR (95% CI) | *P* | HR (95% CI) | *P* |
| ePVS | 1.57 (1.37-1.79) | **<0.001** | 1.29 (1.14-1.46) | **<0.001** |
| ePVS levels |  |  |  |  |
| [4.63-5.79) | Ref |  | Ref |  |
| <4.63 | 0.55 (0.32-0.96) | **0.035** | 0.78 (0.37-1.65) | 0.520 |
| ≥5.79 | 2.36 (1.46-3.81) | **<0.001** | 2.91 (1.36-6.25) | **0.006** |
| Variables | Non-HF  (n=483) | | HF  (n=32) | |
|  | HR (95% CI) | *P* | HR (95% CI) | *P* |
| ePVS | 1.31 (1.22-1.42) | **<0.001** | - | 0.702 |
| ePVS levels |  |  |  |  |
| [4.63-5.79) | Ref |  | Ref |  |
| <4.63 | 0.66 (0.42-1.04) | 0.071 | - | 0.382 |
| ≥5.79 | 2.27 (1.50-3.45) | **<0.001** | - | 0.275 |

The bold numbers represented statistically significant.

ePVS: estimated plasma volume status, HR: hazard ratio, CI: confidence interval, Ref: reference, GCS: the Glasgow Coma Scale, SOFA: the Sequential Organ-Failure Assessment, AKI: acute kidney injury, HF: heart failure.

Model 1: unadjusted model;

Model 2 for age subgroups: adjusted for ICU types, HR, SpO_2_, SOFA, CCI, platelet, glucose, AG, urine output, mechanical ventilation, vasopressors, mannitol and β-blockers;

Model 2 for SOFA subgroups: adjusted for age, ICU types, HR, SpO_2_, CCI, platelet, glucose, AG, urine output, mechanical ventilation, vasopressors, mannitol and β-blockers;

Model 2 for GCS, AKI, sepsis and HF subgroups: adjusted for age, ICU types, HR, SpO_2_, SOFA, CCI, platelet, glucose, AG, urine output, mechanical ventilation, vasopressors, mannitol and β-blockers.

Table S7. Association between ePVS and 1-year mortality in different subgroups for HIS

| Variables | Age <60  (n=200) | | Age ≥60  (n=315) | |
| --- | --- | --- | --- | --- |
|  | HR (95% CI) | *P* | HR (95% CI) | *P* |
| ePVS | 1.25 (1.14-1.38) | **<0.001** | 1.32 (1.17-1.48) | **<0.001** |
| ePVS levels |  |  |  |  |
| [4.63-5.79) | Ref |  | Ref |  |
| <4.63 | 1.35 (0.84-2.17) | 0.212 | 0.92 (0.69-1.38) | 0.872 |
| ≥5.79 | 3.59 (2.14-6.01) | **<0.001** | 2.40 (1.65-3.49) | **<0.001** |
| Variables | GCS <13  (n=221) | | GCS ≥13  (n=294) | |
|  | HR (95% CI) | *P* | HR (95% CI) | *P* |
| ePVS | 1.15 (1.06-1.25) | **0.001** | 1.35 (1.16-1.58) | **<0.001** |
| ePVS levels |  |  |  |  |
| [4.63-5.79) | Ref |  | Ref |  |
| <4.63 | 0.79 (0.55-1.14) | 0.206 | 1.79 (1.13-2.83) | **0.012** |
| ≥5.79 | 1.67 (1.14-2.41) | **0.008** | 4.57 (2.73-7.65) | **<0.001** |
| Variables | SOFA <1  (n=205) | | SOFA ≥1  (n=310) | |
|  | HR (95% CI) | *P* | HR (95% CI) | *P* |
| ePVS | 1.34 (1.15-1.58) | **<0.001** | 1.03 (0.98-1.08) | 0.209 |
| ePVS levels |  |  |  |  |
| [4.63-5.79) | Ref |  | Ref |  |
| <4.63 | 1.10 (0.73-1.68) | 0.642 | 1.11 (0.76-1.62) | 0.583 |
| ≥5.79 | 2.63 (1.63-4.25) | **<0.001** | 2.85 (1.92-4.25) | **<0.001** |
| Variables | Non-AKI  (n=471) | | AKI  (n=44) | |
|  | HR (95% CI) | *P* | HR (95% CI) | *P* |
| ePVS | 1.27 (1.18-1.36) | **<0.001** | 1.49 (0.96-2.30) | 0.073 |
| ePVS levels |  |  |  |  |
| [4.63-5.79) | Ref |  | Ref |  |
| <4.63 | 1.06 (0.80-1.42) | 0.683 | 2.23 (0.53-9.37) | 0.274 |
| ≥5.79 | 2.82 (2.05-3.88) | **<0.001** | 5.72 (1.19-27.50) | **0.030** |
| Variables | Non-sepsis  (n=378) | | Sepsis  (n=137) | |
|  | HR (95% CI) | *P* | HR (95% CI) | *P* |
| ePVS | 1.31 (1.18-1.46) | **<0.001** | 1.21 (1.10-1.33) | <0.001 |
| ePVS levels |  |  |  |  |
| [4.63-5.79) | Ref |  | Ref |  |
| <4.63 | 1.19 (0.87-1.65) | 0.278 | 0.82 (0.47-1.45) | 0.497 |
| ≥5.79 | 2.67 (1.87-3.82) | **<0.001** | 2.82 (1.59-4.99) | **<0.001** |
| Variables | Non-HF  (n=483) | | HF  (n=32) | |
|  | HR (95% CI) | *P* | HR (95% CI) | *P* |
| ePVS | 1.26 (1.17-1.35) | **<0.001** | 1.53 (0.79-2.97) | 0.206 |
| ePVS levels |  |  |  |  |
| [4.63-5.79) | Ref |  | Ref |  |
| <4.63 | 1.08 (0.81-1.44) | 0.591 | 1.45 (0.24-8.71) | 0.685 |
| ≥5.79 | 2.73 (2.00-3.73) | **<0.001** | 5.94 (0.35-101.551) | 0.219 |

The bold numbers represented statistically significant.

ePVS: estimated plasma volume status, HR: hazard ratio, CI: confidence interval, Ref: reference, GCS: the Glasgow Coma Scale, SOFA: the Sequential Organ-Failure Assessment, AKI: acute kidney injury, HF: heart failure.

The subgroup analysis adjusted for age, ICU types, HR, SpO_2_, SOFA, CCI, platelet, glucose, AG, brain surgery, mechanical ventilation, vasopressors, mannitol and transfusion, but excluded subgroup variables.
